# The Aryl Hydrocarbon Receptor Meets Immunology: Friend or Foe? A Little of Both

**DOI:** 10.3389/fimmu.2014.00458

**Published:** 2014-10-02

**Authors:** Walker Julliard, John H. Fechner, Joshua D. Mezrich

**Affiliations:** ^1^Division of Transplantation, Department of Surgery, University of Wisconsin School of Medicine and Public Health, Madison, WI, USA

**Keywords:** indoleamine 2,3-dioxygenase, Th17 Cells, Treg cells, immunomodulation, kynurenine, aryl hydrocarbon receptor

## Abstract

The aryl hydrocarbon receptor (AHR) has long been studied by toxicologists as a ligand-activated transcription factor that is activated by dioxin and other environmental pollutants such as polycyclic aromatic hydrocarbons (PAHs). The hallmark of AHR activation is the upregulation of the cytochrome P450 enzymes that metabolize many of these toxic compounds. However, recent findings demonstrate that both exogenous and endogenous AHR ligands can alter innate and adaptive immune responses including effects on T-cell differentiation. Kynurenine, a tryptophan breakdown product, is one such endogenous ligand of the AHR. Expression of indoleamine 2,3-dioxygenase by dendritic cells causes accumulation of kynurenine and results in subsequent tolerogenic effects including increased regulatory T-cell activity. At the same time, PAHs found in pollution enhance Th17 differentiation in the lungs of exposed mice via the AHR. In this perspective, we will discuss the importance of the AHR in the immune system and the role this might play in normal physiology and response to disease.

## Introduction

Our laboratory has been actively investigating the role of the aryl hydrocarbon receptor (AHR) in the immune system, and the variable effects seen after binding endogenous and exogenous ligands. Study of this receptor traditionally was in the domain of toxicologists, as it was originally defined as a receptor to 2,3,7,8-tetrachlorodibenzo-*p*-dioxin (TCDD) (1). It was further identified that additional toxins, including polycyclic aromatic hydrocarbons (PAHs), also bind to the AHR (2–4). As the mechanism of AHR activation and function was characterized, the importance of this receptor as a transcription factor was realized. The AHR is a cytosolic receptor that after binding migrates to the nucleus where it becomes a transcription factor for cytochrome P450 genes that encode enzymes that metabolize toxins, including those same toxins that bind to the receptor (5). A connection to the immune system had been previously recognized, primarily based on the knowledge that exposure to TCDD led to rapid thymic involution, and animals and human beings exposed to TCDD were known to be immunosuppressed. Effects of TCDD on dendritic cells (DCs) and T-cells have been recognized for years (6), and generation of regulatory T-cells by an AHR-dependent mechanism was identified in 2005 (7). But in general, immunologists and those studying autoimmunity did not become interested in the AHR until 2008, when two high impact papers identified the role of the AHR in T-cell differentiation, with certain ligands enhancing Treg generation, and others enhancing Th17 differentiation, both *in vitro* and *in vivo* (8, 9). Since that time, there has been a flood of reports on the role of the AHR in the adaptive immune system and animal models of disease, particularly autoimmune disease (10–12).

The role of the AHR in responding to toxins is thought to have evolved, as invertebrate forms of the AHR do not bind TCDD (13), and the toxins associated with the AHR are man made and were generated long after the origins of the receptor. It has been postulated that the initial importance of the AHR was in embryologic development, and in addition there was (and remains) a requirement for binding of this receptor to endogenous ligands early in development. This is based on the abnormal phenotype of AHR null mice that display a patent ductus venosus (14), as well as the necessity for the hypomorphs to bind AHR ligand early in development to prevent the patent ductus venosus in these transgenic mice (15). In addition to binding to pollution and endogenous ligands, the AHR binds to numerous ligands present in the diet including flavonoids, which are ubiquitous in fruits and vegetables. There has been an ongoing search for relevant endogenous ligands in animals and human beings.

**Figure 1:**
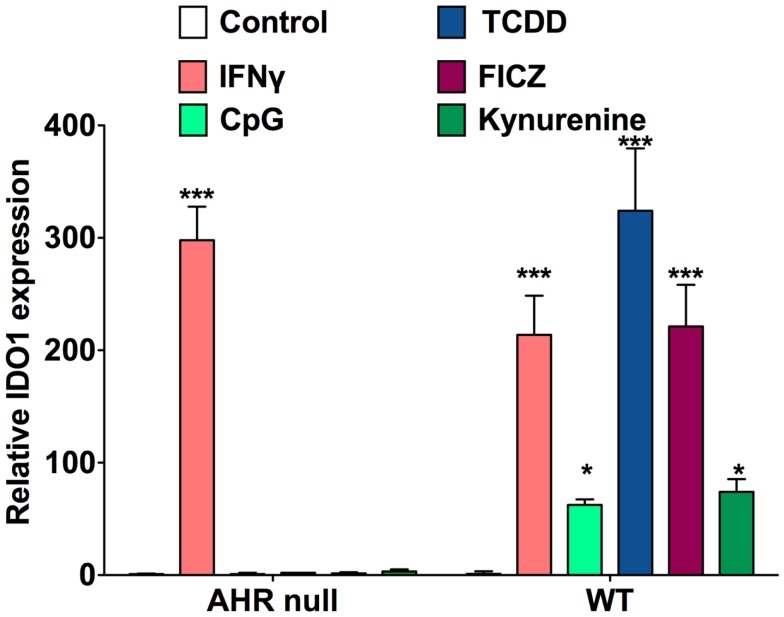
**AHR ligands increase IDO_1_ expression in BMDCs**. qRT-PCR analysis of IDO_1_ mRNA expression in bone marrow-derived DCs treated for 48 h with IFN-γ (10 μg/ml), CpG (2.5 μM), TCDD (10 nM), FICZ (200 nM), or kynurenine (50 μM). Data are from three independent experiments.

Around the time that the AHR was first recognized to play some role in the acquired immune system by toxicologists, immunologists were investigating indoleamine 2,3-dioxygenase (IDO_1_), initially recognized as the rate-limiting enzyme in tryptophan metabolism. In 1998, it was reported that tryptophan catabolism by IDO_1_ and other enzymes was responsible for prevention of allogeneic fetal rejection in mice, and it was further revealed that this enzyme was generated by DCs, and in some way increased the differentiation of Tregs (16). The mechanism was unclear, but the two leading theories were that either tryptophan deprivation from its metabolism decreased the generation and division of effector cells (17), or certain breakdown products of tryptophan, termed kynurenines, were acting through some target on T-cells or other cells to favor Treg differentiation (18, 19). In our own lab, we considered the hypothesis that kynurenine or one of its breakdown products was working through the AHR in T-cells to enhance Treg generation. This was based on the knowledge that indoles and other tryptophan derivatives are often ligands of this receptor, making kynurenine a good candidate as an endogenous ligand. We identified that not only does kynurenine bind to the AHR in the cytosol of T-cells and enhance Treg generation *in vitro* but additionally kynurenine and other AHR ligands act on BMDCs to upregulate IDO expression in an AHR-dependent manner (20) (Figure 1). The effects of AHR ligands on differentiation and function of natural versus induced Tregs has not yet been delineated.

One of the controversial and yet unanswered questions about the role of the AHR in the immune system is whether it is probable that different ligands bind to the AHR and lead to different, almost opposite outcomes, perhaps based on some conformational change or transcription of different genes, a finding that would almost be unprecedented in physiology. We theorize that some combination of strength of binding, duration of binding, route of exposure, and surrounding milieu determines whether Treg or Th17 differentiation is enhanced. Although early experience seemed to suggest that ligands were either “regulatory” (meaning they favored differentiation of Tregs) or “effector” (meaning they favored differentiation of effector Th17 cells) (8), more recent studies and our own experience has suggested that this is not likely the case. For instance, the ligand 6-formylindolo[3,2-b]carbazole (FICZ) was first described as enhancing Th17 differentiation *in vitro* and *in vivo*, and when administered subcutaneously worsened autoimmunity in an experimental autoimmune encephalomyelitis (EAE) model in mice. However, it has recently been shown that when this same ligand is administered intraperitoneally, Treg differentiation is favored and there is a reduction in EAE disease severity (21, 22). Our own experience has also shown that depending on the culture conditions or inflammatory status of the animals, similar ligands can enhance either Th17 or Treg generation. We have also found that every ligand we have tested stimulates T-cells to generate IL-22, and it has been demonstrated that the AHR is required for IL-22 generation in the majority of scenarios (23–25). IL-22 is an interesting cytokine that is generated by various types of T-cells, but its receptor is found on epithelial cells. In general, IL-22 is thought to be protective against immune damage and helpful in epithelial cell repair (26–28). We have recently shown that particulate matter (PM) in the form of urban dust particles (UDP) from the National Institute of Standards and Technology (NIST; SRM1649b), containing PAHs, increases the Th17 response both in the lung *in vivo* and in T-cells *in vitro* (29). The *in vitro* response was shown to be AHR dependent. Therefore, a connection between the AHR and the immune system seems real, but the effects are complicated and dependent on the model or conditions being examined. This relationship of the AHR with the immune system, where binding of this receptor with different ligands in differing milieus can lead to seemingly opposing responses in T-cell differentiation has allowed us to come up with the following hypothesis:

## The AHR as a Sensor

**Figure 2:**
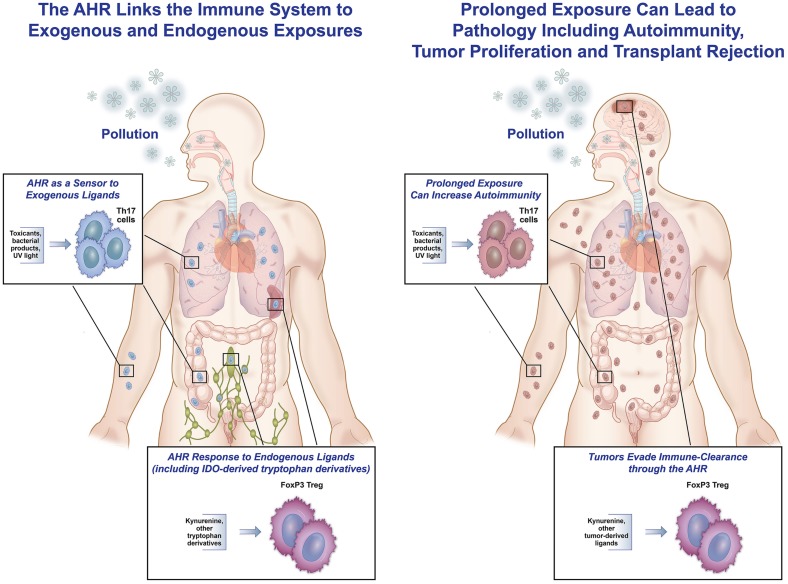
**The AHR as friend and foe**. This figure represents the hypothesis that the AHR can be both protective and pathologic in responding to endogenous and exogenous ligands. **On the left**, exposure to inhaled pollution (as well as ingested or topical) can bind to the AHR in immune cells, and in an inflammatory setting, lead to increased Th17 differentiation, IL-22 generation, and cytochrome P450 enzyme production. All of these responses can help protect the host from pathologic inflammation and invasion by endogenous bacteria. Similarly, in response to endogenous ligands during inflammation (in the spleen, lymph node, or throughout the body), AHR ligands can lead to increased IDO production and differentiation of Tregs by binding of kynurenine to the AHR in T-cells, hence controlling an immune response. **On the right**, examples of binding of the AHR leading to increased pathology are shown. Tumors (in the brain or elsewhere) have been shown to generate TDO, leading to kynurenine production that binds to the AHR in tumor cells, causing increased regulation and decreased tumor destruction by the native immune system. In addition, prolonged exposure to external pollutants can lead to over-differentiation of Th17 cells and aggravation of autoimmunity in the lungs, gut, skin, and elsewhere in susceptible people.

The AHR serves as a sensor that responds to signals, both from the outside environment or internal milieu, to modulate an immune response. In the setting of an endogenous immune response, the AHR helps contain the inflammation and decreases collateral damage, so the response does not cascade in strength and lead to autoimmune destruction. In responding to exogenous ligands, the AHR functions as a sensor to “danger” signals, such as dioxins, PAHs, and other components of pollution. This is of evolutionary benefit for individuals exposed to man-made pollution or other toxins, as illustrated by the following scenario. When someone is exposed to inhaled atmospheric pollution, damage to bacterial clearance mechanisms and the epithelial lining of the airway puts the individual at risk for endogenous bacterial invasion and activation of a reactive airway response. The AHR present in immune cells residing in the lung binds to fractions of PM (PAHs and others), leading to an enhancement of Th17 differentiation that can serve as an immune barrier to the endogenous bacteria found in the lining of the lung. Simultaneously, AHR activation leads to generation of IL-22 that helps with epithelial cell repair and maintenance of tight junctions. Finally, activation of the AHR causes an upregulation of cytochrome P450 enzymes that metabolize the harmful toxicants. Taken together, the AHR aids in tissue protection, repair, and toxin clearance (Figure 2, left).

### Endogenous ligands

We hypothesize that as the adaptive immune system developed, it became crucial that there be a way for the body to dampen overly robust responses, thereby preventing reactivity to self-proteins and ensuing autoimmunity. Kynurenine, the first breakdown product of tryptophan in the IDO pathway, became the transmitter that interacts with the AHR in T-cells and enhances the differentiation of Tregs that reduce an immune response. At the same time, kynurenine also interacts with the AHR in DCs to enhance the generation of IDO, leading to further tryptophan break down. This response is self-limited, as once the tryptophan in the inflammatory milieu is dissipated, kynurenine is no longer generated and Treg generation is decreased. This push toward a regulatory response from endogenous ligands makes evolutionary sense, as internal inflammatory responses would need to be controlled to prevent damage to surrounding epithelial and parenchymal cells at the sight of an immune response. Additionally, binding of the AHR to ligand leads to the generation of IL-22 and engenders epithelial cell homeostasis, proliferation, and anti-microbial peptide production (30), further protecting the bystander tissue.

### Exogenous ligands

In contrast to the role that the AHR plays in regulating autoimmunity when presented with endogenous ligands, at interface organs such as the lung, gut, and skin, AHR activation leads to an effector response. At these locations, toxin binding to the AHR in immune cells leads to Th17 differentiation and a heightened inflammatory response. We view this interaction as a “danger” signal that allows activation of the immune system at the same time that P450 enzymes are upregulated to metabolize toxins. When a host is presented with a potentially harmful exposure, early signaling and activation of the immune system occurs concurrently with ligand clearance. We believe that this is the role of exogenous activation of the AHR in immune cells.

It is important to again point out that we do not believe that endogenous ligands such as kynurenine always favor Treg generation, and exogenous ligands including fractions of pollution always favor Th17 differentiation. However, we do believe that in the *in vivo* inflammatory milieu where exogenous ligands are exposed to interface organs during a toxic environmental exposure, Th17 differentiation is favored, and similarly in the endogenous *in vivo* milieu of an immune response in the presence of IDO and tryptophan, Treg generation is favored. The mechanistic details of these effects on T-cell differentiation require further delineation and are an exciting area of research.

## Pathologic Response

While we hypothesize that the AHR and its role in the immune system evolved to be protective, there are examples where this interaction actually increases pathology. In the setting of an overwhelming exposure to exogenous ligands, generation of ligands by tumors, or changes in the microbiome altering physiologic interactions between products of bacteria and receptors in the gut, the AHR can worsen pathology (Figure 2, right).

### Cancer

Certain cancers have developed the ability to evade their own clearance through use of the AHR. In gliomas, constitutive activation of tryptophan 2,3-dioxygenase (TDO-2) leads to high levels of kynurenine production. The TDO-2-derived kynurenine then acts through the AHR in tumor cells in an autocrine manner and promotes tumor survival as well as altering the regional immune response by favoring Treg development. These Tregs hamper the ability of the immune system to clear the tumor. This is particularly effective as the interaction between kynurenine and T-cells in the vicinity of the tumor leads to local regulation in the very location and milieu that the tumor resides in Ref. (31). In addition, there are some examples in human cancer where the tumors show increased copies of the AHR (32), which could increase the autocrine effect of preventing tumor clearance.

### Autoimmunity

The immune system is in a constant balance of regulation and inflammation, and loss of this equilibrium can lead to pathology. We hypothesize that exogenous ligands of the AHR found in pollution can aggravate autoimmune conditions in an AHR-dependent mechanism. For many years, there has been speculation that environmental exposures can aggravate or even cause autoimmune disease, and a recent analysis of epidemiologic data has supported this hypothesis. The specific exposures that lead to disease and the mechanisms through which they impart pathology remain unclear. Recent data suggest that modulation of the Treg/Th17 balance may be at the center of environmentally induced autoimmunity. An NIEHS workshop concluded that “dysregulated Th17 activity can lead to pathology” in various autoimmune diseases, and that both smoking and “AHR binding by aromatic hydrocarbons favors differentiation of Th17 cells and can exacerbate autoimmunity” (33). We recently identified that inhaled PM leads to an increase in Th17-driven inflammation (29). In our resultant model, when an individual is exposed to inhaled PM for a prolonged period of time, there is a shift in the Treg/Th17 balance toward Th17 cells, aggravating or initiating autoimmunity in susceptible people. This ubiquitous mechanism will be applicable to multiple environmentally induced autoimmune diseases.

### Transplant rejection

One of the novel theories we have been studying in our lab is the possibility that exposure to pollution is altering the Treg/Th17 balance and leading to chronic rejection after solid organ transplant. This may be most relevant to lung transplantation, where the transplanted organ is at the interface with the outside environment (34). There is growing evidence that inhaled exposure to elevated levels of atmospheric pollution, with increased PM, increases chronic rejection in lung transplants in human beings (35, 36). Bronchiolitic obliterans syndrome (BOS) is the major form of chronic rejection in lung transplantation, and it has been well documented that Th17 cells and the cytokine IL-17 are required for pathology (37–39). Given the link between IL-17 and BOS, the fact that pollution accelerates BOS, and our own work demonstrating that pollutants contain AHR ligands, which favor a Th17 response, it is possible that the AHR is a key factor in pollutant accelerated rejection in lung transplantation.

### Gut immunity

Some of the highest impact papers regarding the AHR in the last couple of years have described the relationship between gut immunity and the AHR. The presence of this receptor is necessary for the establishment of certain populations of immune cells in the gut. Furthermore, both dietary ligands of the AHR as well as ligands produced by gut bacteria interact with and alter the gastrointestinal immune system (40, 41). The healthy microbiome generates ligands that interact with the AHR to maintain gut immune structure and stability. Loss of some of these ligand-producing bacteria may increase the risk of autoimmune colitis (42).

### Skin

The AHR is highly expressed in multiple cell types found in skin. As skin is one of the organs at the interface with the outside environment, it is likely that inflammatory disorders are altered by interaction with toxins and other environmental factors. A recent publication did document the protective role of various AHR ligands in reducing inflammation in a model for psoriasis (22). At the same time, exposure to AHR antagonists increased inflammation. This is clinically relevant, as it is known that certain toxicants contain fractions that may inhibit the AHR or cytochrome P450 enzymes, and certain weak AHR agonists actually inhibit activation by other agonists that may be found in different fractions of a given exposure (43). This implies that in clinically relevant mixtures of chemicals, the overall response could be one of antagonism of the AHR and worsening of skin inflammation.

## AHR Meets Immunology: Friend or Foe?

So does the relationship between the AHR and the immune system make us healthier and more able to withstand interactions with the environment, or leave us at risk for pathologic manipulation of the immune system in response to external and internal signals? We assert that the AHR does make us healthier, and allows us to survive in the environment that currently exists. The relationship of the endogenous ligand kynurenine and the AHR allows a feedback loop that dampens an immune response to prevent too much inflammation and autoimmunity, allowing a response to be self-limited. Exogenous ligands serve as “danger” signals that alert the immune system to toxic insults from the environment, allowing immune cells to play an early role in containing damage done by these exposures, helping repair local barriers, and containing local endogenous infectious exposures. At the same time, there are examples where disease processes have taken advantage of this connection to increase pathology. This scenario, where normal physiology or “protective” responses become overwhelmed and lead to increased severity of illness, is well documented in immune responses to pathology, including systemic inflammatory response syndrome in response to infection, multiple forms of autoimmunity, and resistance of cancer to its own destruction. As we better understand the mechanisms and signals behind the role of the AHR in the immune system, we will be able to manipulate this receptor to treat or prevent diseases as diverse as cancer, autoimmunity, and transplant rejection.

## Conflict of Interest Statement

The authors declare that the research was conducted in the absence of any commercial or financial relationships that could be construed as a potential conflict of interest.
